# Preparedness components of health systems in the Eastern Mediterranean Region for effective responses to dust and sand storms: a systematic review

**DOI:** 10.12688/f1000research.17543.1

**Published:** 2019-02-04

**Authors:** Kiyoumars Allahbakhshi, Davoud Khorasani-Zavareh, Reza Khani Jazani, Zohreh Ghomian

**Affiliations:** 1 Department of Health in Disasters and Emergencies, School of Public Health and Safety, Shahid Beheshti University of Medical Sciences, Tehran, Iran; 2 Clinical Sciences and Education Department, Karolinska Institute, Stockholm, Sweden; 3 Safety Promotion and Injury Prevention Research Center, Shahid Beheshti University of Medical Sciences, Tehran, Iran

**Keywords:** dust and sand storm, health, preparedness, reediness, Eastern Mediterranean Region

## Abstract

**Background:** Dust and Sand Storm (DSS), according to estimates by global reports, will increase dramatically in the Eastern Mediterranean Region (EMR). Numerous health problems caused by DSS will be severely affected regions and vulnerable groups. This study aimed to identify the components of the preparedness of health systems for the DSS phenomenon in EMR.

**Methods: **In this systematic review, the peer-reviewed papers in four electronic databases, including Medline through PubMed, Scopus, ISI Web of Science and the Cochrane library, as well as available grey literature, were searched and selected. The research process was carried out by including papers whose results were related to the potential health effects caused by desert dusts in EMR. Was used the combination of three groups of keywords: the exposure factor, health effects as outcomes, and the countries located in EMR. The focus was on the PRISMA checklist, with no time limitations until December 2017. Finally, through 520 related citations, 30 articles were included. Descriptive and thematic content analyses were evaluated.

**Results:** The preparedness components were divided into three and ten main categories and subcategories, respectively. The three categories covered the areas of DSS hazard identification, planning and policy-making, and risk assessment.

**Conclusions:** Recognition of the health system preparedness factors for DSS in EMR will help policy-makers and managers perform appropriate measures when dealing with this hazard. More studies should be conducted to understand these factors in other parts of the world.

**Registration: **PROSPERO registration number CRD42018093325.

## Introduction

Dust and Sand Storms (DSS), as a natural disaster from the type of meteorological hazards ^1, 2^, affects the atmospheric system, air quality, and human health ^3– 6^. Over the recent years, the review studies have shown that there is a relation between the occurrence of the DSS and the incidence of human health ^7, 8^.

Based on the division by the World Health Organization (WHO), there are 22 countries in the Eastern Mediterranean Region (EMR) ^9^. According to estimates from the Intergovernmental Panel on Climate Change (IPCC) report, EMR is one of the areas that will be strongly affected by this phenomenon in the future and will have potentially harmful health effects, especially on vulnerable groups ^10, 11^. Therefore, the best way to minimize the damage caused by the disasters is the preparedness for them ^12^. The constant increasing risk of hazards indicates the need to integrate disaster risk reduction in preparedness, reinforce disaster preparedness, and provide assurance for timely utilization of the capacities ^13^.

The epidemiological studies conducted on DSS mainly focus on the effects of the phenomenon on mortality and morbidity in humans ^14– 20^ or on the nature of major sources of dust phenomenon, the frequency, the duration and changes to the occurrence of the DSS, and the content of particulate matter (PM) suspended in the air ^8^. Considering the DSS challenges on human health, drastic measures should be taken to ensure the health system is prepared for this phenomenon. In this regard, important elements have been proposed in the health system preparedness for disasters including the identification of hazards, the provision of Emergency Operation Plan (EOP), training and education, equipment, Early Warning System (EWS), information exchange, drill and exercise, monitoring, and evaluation ^13, 21– 28^. On the other hand, there has not been a comprehensive study on the factors affecting the preparedness of the health system for dust phenomenon in EMR. Accordingly, the present study, through systematic review (SR), aims to investigate the factors affect the health system preparedness for the DSS in EMR and identify research gaps on the preparedness of the health system for DSS. The findings of the study, with proposed recommendations, can directly help health policymakers in preparedness promotion for this phenomenon, pave the way for further studies, and add to the richness of the current knowledge.

## Methods

This study reports a SR based on the recommendations of the Cochrane and PRISMA guideline ^29^. The PROSPERO registration number is CRD42018093325. A completed PRISMA checklist is available on figshare ^30^.

### Inclusion and exclusion criteria

All English-language articles related to effective factors on the preparedness of the health system for dust phenomenon in EMR were searched until December 2017. All methods of study, books, and theses that associated with the subject of this research were included. Papers in the form of the letter to the editor and studies that were merely related to the dust subject, and in which no mention of health outcome, were excluded, as were non-English-language articles, as were those without full-text access. Additionally, scientific documents related to non-desert origin (such as volcanic or anthropogenic sources) were not included.

### Data sources

The international electronic databases investigated by authors (KA, ZGH, DKZ), including English sources from Medline through PubMed, Scopus, ISI web of science, Google Scholar, Cochrane library. The Eastern Mediterranean Health Journal and African Journals OnLine were also searched for published articles in the EMR. Other documents were extracted from reports published by organizations (such as the United Nations).

### Search strategy

**Figure 1.**
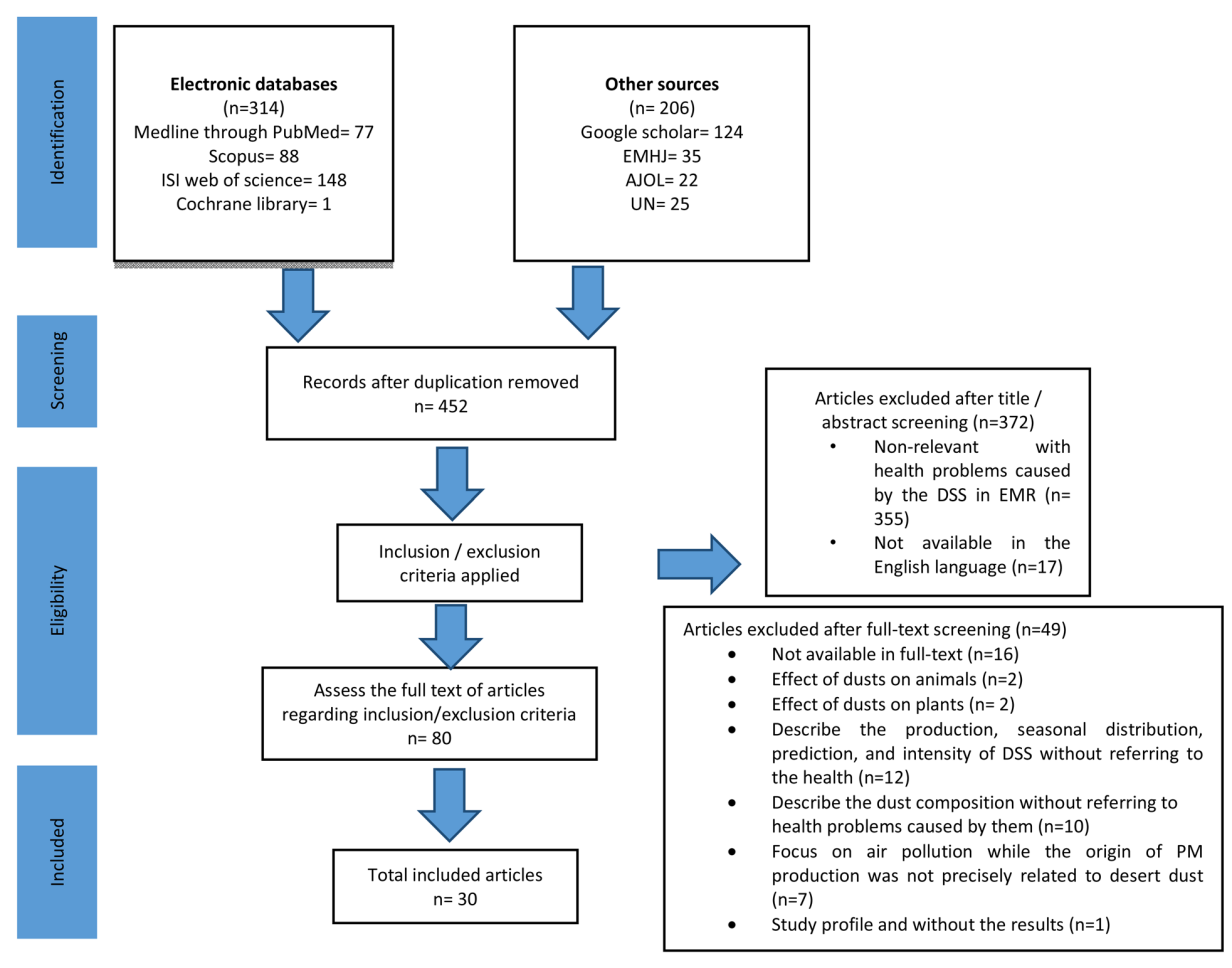
PRISMA flowchart to extract effective components on the preparedness of the health systems for DSS in the EMR until December 2017.

Following consultations with Library & Information Science (LIS) professionals in the health field, the search for articles was done using a combination of three groups of words in the databases mentioned above: The exposure factor (Dust Storm OR Sand Storm OR desert dust), health effects as outcomes (health OR morbidity OR mortality OR disease OR hospital OR respiratory OR cardiovascular OR coccidiomycosis OR meningococcal meningitis OR conjunctivitis OR dermatological OR transport accidents), and member states of the EMR (Middle East OR Afghanistan OR Bahrain OR Djibouti OR Egypt OR Iran OR Iraq OR Jordan OR Kuwait OR Lebanon OR Libyan Arab Jamahiriya OR Morocco OR Oman OR Pakistan OR Occupied Palestinian Territory OR Qatar OR Saudi Arabia OR Somalia OR Sudan OR Syrian Arab Republic OR Tunisia OR United Arab Emirates OR Yemen). These three groups of words were combined with "AND" together. The keywords were obtained from previous articles relating to the challenges of the health domain in connection with the DSS. In order to increase the probability of identification of all relevant literature, these keywords were selected based on an agreement of three researchers (KA, ZGH, and DKZ). The search words are used in titles, abstracts, and keywords of used articles. Figure 1 shows the search strategy.

### Data collection process

In order to manage the citations, EndNote software, version X15, was used. All duplicate records became clear and removed. Using the title, abstract and, keywords screening, and given the inclusion criteria, the evaluation of documents was performed by two researchers (KA and ZGH). In the next step, the full text of the remaining articles was analyzed independently by the two researchers considering the inclusion and exclusion criteria and standard quality assessment. The papers were later examined in order to observe the points of the criteria for inclusion/exclusion by two other scholars (DKZ and RKJ). The quality of all articles was evaluated using the Cochrane handbook to evaluate the bias ( Table 1) ^31^. In this SR, the data extraction sheet was designed in two main parts. The first section covers the general specification of articles containing the ID, hyperlinks, title, electronic database, first author, publication year, country or region, method, population, and year/years of study. The second part was used to identify the main findings of articles, research suggestions related to this SR, and the preparedness components of the health system for the DSS. All extracted data were evaluated by members of the research team to verify accuracy and completeness.

**Table 1.** Different types of reporting biases in the SR of preparedness components of health systems in the EMR for effective response in DSS.

Type of reporting bias	Bias assessment across studies were done in this SR
Publication bias	During the quality assessment, checked that have included two abstracts of the articles ^32, 33^ presented at conferences, which have been published fully later. In this SR grey literatures (n= 206) and peer review articles (n = 314) included in the study. Were also considered studies with positive (n=27) and negative (n=5) outcomes. Two articles have both outcomes. It is important to note that access to full texts of some articles was limited (n = 16); however, after reading the abstract the research team found that most of them did not have related information about the aim of our systematic review.
Time lag bias	For positive and negative outcomes, the average per month, between submissions to published, was investigated that it took 4.5 and 3, respectively. However, in this process, different factors can play a role.
Multiple (duplicate) publication bias	The inclusion of multiple publications of the same study was checked within this SR; eventually, and no case found.
Location bias	The citations published in the journals, with various Impact Factors (IF) (non – IF to IF= 4.61), were evaluated. The studies with IF is less than one and non - IF as well as the articles with IF more than one were (n = 10, 33 %) and (n= 20, 67 %), respectively. The purpose of this study was concentrated primarily on the EMR. Therefore, Only articles in this area were reviewed.
Citation bias	In this SR, the research team tried to take into account the inclusion criteria when using other related references. Also, were included both positive and negative outcomes to the SR.
Language bias	One of the inclusion criteria for this SR was the English language. We did not have the possibility of translating other non - English languages, and this was the limitation of the study.
Outcome reporting bias	Given the objective of this SR, there was no possibility of exploring this type of bias. No RCT included to the SR.

## Results

In general, through the implementation of the research strategy, the number of 520 records was found. In the last stage, 30 unique articles were obtained based on the inclusion/exclusion criteria ( Figure 1). Data on the risk evaluation of bias are provided in Table 1. Final articles were examined in two parts, including descriptive and thematic content analysis. In Table 2, more details are presented about the imported literature to the SR. Findings indicate that since 2008, researchers have published more articles about the effects of the DSS on public health. Most articles related to Iran (17 articles, 56%). None of them had a qualitative approach. In thematic content analysis, based on literature review of studies that identify factors affecting the health system preparedness for various hazards ^21– 28^ and the multistage analysis by the research group, the preparedness components of health system for DSS in EMR were extracted and classified ( Table 3).

**Table 2.** Selected articles in the SR on the factors affecting the preparedness of the health systems for the DSS in the EMR.

Related reference	First author, year	Country	District/national/ international/ level	Study design	Population	Main outcomes
34	Geravandi S., 2017	Iran	District	Descriptive study	data of hospital admission respiratory patients and dusty days	Hospital admission cases were significantly higher during the dusty days compared to other days (p = 0.0002).
47	Shabankarehfard E., 2017	Iran	District	matched case-control study	45 cases of allergic patients and 45 in the control group	Penicillium and Alternaria (available in outdoor dusts), as well as Aspergillus flavus and Cladosporium (available in indoor dusts), cause allergic diseases. On the other hand, the Alternaria in the homes has a significant adverse association with asthma prevalence.
35	Naimabadi A., 2016	Iran	District	Cross-sectional study/ laboratory study	15 PM10 filters for normal days and 15 PM10 filters in the dusty days	In dusty days, the increase of the metal concentration in the dust was more than other days.
36	Neisi A., 2017	Iran	District	Cross-sectional study	110 non-asthmatic elementary school students	Researchers by comparing the Fractional exhaled nitric oxide (FENO) and pulmonary functions as parameters of adverse health effects caused by PM have found that there is a significant association between PM concentration and FENO in healthy students.
57	Hosseini G., 2015	Iran	District	Cross-sectional study/ laboratory analysis	The number of samples belonging to non - dusty days and dusty days were 44 cases and 9 cases, respectively.	During the dusty days, the main elements contained in PM10 included (Na, Ca, Mg, Al, Fe).
45	Ebrahimi S. J. A., 2014	Iran	District	Cross-sectional study	The data associated with the PM10 concentration and air quality as well as the data from the cardiovascular and respiratory disease associated with dust	The PM10 concentration for every 100 μg \ m ^3^, was found to be the significant statistical correlation with (cardiovascular disease) and non - significant statistical correlation with (respiratory problems).
37	Khaniabadi Y. O., 2017	Iran	District	Cross-sectional study	PM10 samples obtained from air pollution monitoring stations	4.7% and 4.2% of the hospital admission cases were the cardiovascular and respiratory patients associated with the PM10 concentration of up to 10 μg \ m ^3^, respectively.
38	Amarloei A., 2015	Iran	District	Cross-sectional study	Selection of 250 persons and in the next stage random cluster sampling from 13 health centers	According to the values of breathing capacities, 21.6% of the participants suffered from obstructive lesions. It was in men more than women.
49	Moradian M.J., 2015	Iran	District	brief incident report	Citizens of Tehran	In general, a total of 5 deaths and 44 injuries caused by the dust storm. Other damages included floating debris, knocked down trees, car accident, temporary shutdown of (electricity, Internet and telephone), delay in flights and damage to the structures.
55	Nourmoradi H., 2015	Iran	District	Cross-sectional study/ laboratory analysis	PM10's daily sampling was carried out at a site of the city. Every 7 days and dusty days, Microbial sampling has been performed.	The PM10 concentration in May, June, and July (twice a month) was higher than standard values. The average number of bacteria and fungi in the air during the dusty days was (1.5 and 3.83 times) the non-dusty days. During the dust period, the most abundant type of bacteria and fungi observed in the samples, respectively, are due to (Bacillus spp and Mycosporium spp.).
39	Thalib L., 2012	Kuwait	National	population-based retrospective time series study	15256 emergency asthma and respiratory patient as well as PM10 data from 6 air monitoring stations.	Around 33.6% of the days was the dust storm (PM10> 200 μg / m ^3^). These days significantly related to an increase in emergency admission of asthmatic and respiratory patients on the same day. Based on the findings, were about 4% to 8% of all respiratory cases associated with DSS, most of which were children.
18	Al-Taiar A., 2014	Kuwait	National	population based retrospective ecological time series study	death cases and data of dusty days and air pollution (569 days) of 6 air pollution monitoring stations	There was no statistically significant correlation between the occurrence of DSS and respiratory death.
44	Bener A., 1996	UAE	District	Cross-sectional study	850 school children (6 to 14 years)	DSS cause asthma among students.
40	Marzouni M.B., 2016	Iran	District	Cross-sectional study	PM10 hourly data	7.6, 11, 15.1, 13.5, and 7.6% of TM, CM, RM, HARD, and HACD were related to short-term exposure with PM10.
46	Soleimani Z., 2013	Iran	District	Cross-sectional study	Sampling from three sites (school, university, and hospital) in three seasons, 264 times during the non-dusty days and 66 times in the dust days, was carried out.	The concentration of fungi (Cladosporium, Alternaria, Aspergillus, Penicillium, Rhizopus) during the dusty days was more than the other days. Aspergillus was dominant in summer and autumn and Cladosporium in the winter. In the morning, the high concentration of fungus was observed.
32	Miri A., 2007	Iran	District	Cross sectional study	Synoptic stations weather data and 150 questionnaires	The main problem of patients in dust days is respiratory disorders (COPD and asthma). These respiratory problems brought economic losses to about US $ 66 million.
33	Meibodi A.E., 2015	Iran and Iraq	Regional	Mixed method (literature survey and cooperative games theory and Shapley solution)	NA	The total estimated losses from DSS to the health system in Iran and Iraq amounted to the US $306 million, respectively. The total losses in the two countries were estimated at the US $1043 and the US $1404 million, respectively.
50	Almasi A., 2016	Iran	District	Cross-sectional study	PM10 concentration data and death data due to traffic accidents	By increasing the dust concentration were reduced traffic accidents.
41	Goudarzi G., 2017	Iran	District	Cross sectional study	PM10 samples obtained from air pollution stations	The number of excess cases estimated to respiratory death, hospital admission of COPD patients, respiratory and cardiovascular diseases attributed to the PM10 concentration, were 37, 39, 476 and 184, respectively. Also, 92% of the deaths and disease cases occurred in the days when the concentration of PM10 was less than 150 μg / m ^3^.
56	Soleimani Z., 2016	Iran	District	Cross sectional study	From different parts of the hospital, measured samples of air.	Dominant bacteria in open and closed environments during dusty days and other days were (Bacillus spp., Micrococcus spp., Streptomyces spp., and Staphylococcus spp.).
42	Al-Dabbas M.A., 2012	Iraq	Provincial	Cross sectional study	The dust samples collected from 8 cases of DSS as well as the weather data of 44 stations.	The most common types of bacteria were Bacillus and E.Coli, respectively. Also, the most common types of fungi were Aspergillus and C. Albicans, respectively. In the samples, no viruses were found. The most important pollens were Chenopodiaceous and Graminea.
52	Metcalf J., 2012	Qatar	Provincial	Cross sectional study/ Laboratory analysis	soil samples weighing one kilogram	The existence of microcystins and potentially anatoxin-a (S) in the desert pollens have effects on human health.
51	Richer R., 2015	Qatar	District	Cross sectional study	Examples of desert soil	In the impact of human exposure and inhalation of contaminated dust with Cyanobacterial toxins, health consequences can be found.
48	Alghamdi M.A., 2016	Saudi Arabia	District	Cross sectional study/ Laboratory analysis	Samples of environmental PM on the sampling site	Fe and Al were the most dominant metal in the PM10. The values of Ni and Cd were higher than global standards. The results of the study showed that 108.77 out of anyone million people are at risk of cancer after exposure to carcinogenic heavy metal in the environment.
53	Alangari A.A., 2015	Saudi Arabia	District	Cross sectional study	Children aged 2 – 12, referral to hospital	No correlation between the daily average (PM10 and PM2. 5) and the average number of asthmatic children did not find.
68	Ardalan A., 2016	Iran	National	Cross sectional study	421 hospital in the country	As the first ten hazards in hospitals, according to HSI assessment in 2015, DSS (59%) after the earthquake (71%), and high temperatures (64%) were identified.
66	Ardalan A., 2014	Iran	National	Cross sectional study	224 hospitals nationwide	According to HSI, disasters with the highest probability of occurrence and effects were the earthquake (68%), DSS (62%), and high temperature (58%).
64	Ardalan A., 2013	Iran	National	Retrospective survey	1401 PHC at the country	Among all types of natural hazards, the frequency of DSS and its effects on PHC was: Hazard frequency (2.5%), the impact on health workers (1.6%), the structural damage (0.9%), non - structural damage (0.7%), and functional damage (6.1%).
17	Khaniabadi Y.O., 2017	Iran	District	Ecological study	Air pollution data	4.9% of the hospital visits for COPD and 7.3% of the RM attributed to an increase of 10 μg/m ^3^ of PM10 concentration. There is a direct relationship between increasing PM10 concentrations, to more than 200 μg /m ^3^, at the time of MED and the hospitalization rate.
65	Shaw R., 2016	Iran	National	Country status report and case study	NA	Establishment of DuSNIFF is an initiative measure in the prediction of DSS.

TM, Total Mortality; CM, Cardiovascular Mortality; RM, Respiratory Mortality; HARD, Hospital Admission for Respiratory Diseases; HACD, Hospital Admission for Cardiovascular Diseases; PHC, Primary Health Centers; DuSNIFF, Dust Storm Network-based Integrated System of Forecast and Forewarning.

**Table 3.** Preparedness components of health systems for DSS in EMR.

Main category	Category	Subcategory	Extracted from		Citation
			Study results	Study recommendations	
DSS preparedness components	DSS Hazard identification	Health problems caused by DSS	*	N.A	17, 18, 34– 54
		Composition of DSS	*	N.A	35, 42, 46– 48, 51, 52, 55– 59
		Economic losses on health	*	N.A	32, 33
	Planning and policymaking	Education and training	*	*	17, 36, 49, 60
		Research	N.A	*	18, 35, 37, 39, 43, 52– 55, 57, 61
		Compilation of new and local indexes of air quality	N.A	*	61
		Comprehensive database development	N.A	*	57, 64
		Prediction and warning	*	*	49, 53, 65
	Risk Assessment	Use of hospital safety assessment tools	N.A	*	66, 68
		Use models for health risk assessment of DSS	N.A	*	18, 39, 69

NA, not applicable.

### DSS hazard identification

***Health problems caused by DSS.*** Studies conducted in EMR showed that the prevalence of respiratory diseases (RD) ^17, 34– 43^ and respiratory mortality (RM) is directly related to DSS ^17, 40, 41^. Some of these studies focused on the incidence of asthma and pulmonary dysfunction in schoolchildren ^44^. On the other hand, dust storms result in hospitalization due to cardiac diseases ^37, 40, 41, 45^ and cardiac mortalities (CM) ^40^. Some of the various fungal species in EMR dust storms cause infectious diseases in human beings ^46^ that increase or decrease allergic and asthma diseases ^47^. Exposure to carcinogenic metals in PM10 increases the risk of cancer in citizens ^48^. Other problems reported by the researchers were death and trauma due to the occurrence of storms (at a speed of 110 km/h) with dust particles ^49^. Researchers found that as the concentration of DSS increases, the death caused by road traffic accidents (RTAs) decreases ^50^. Studies conducted in Qatar showed that inhaling cyanotoxins found in DSS could have devastating effects on human health ^51, 52^. In contrast to these results, a number of researchers found that in general DSS had no effect on asthma, RD, RM ^18, 45, 53^, CM ^18^, and RTAs ^54^.

***Composition of DSS.*** The most abundant of bacteria observed in the dust particles were *Bacillus* spp. ^42, 55, 56^, *Staphylococcus* spp., *Streptomyces* spp., *Micrococcus* spp. ^56^ and *Escherichia coli* ^42^. Also, most types of fungi were ( *Mycosporium* spp. ^55^ *Penicillium*, *Aspergillus flavus*, *Cladosporium*, *Alternaria*, *Rhizopus*, and *Cladosporium*) ^46, 47^, *C. albicans* ^42^. The pollen grains caused allergies, identified from dust storms, in descending order, included *Chenopodiaceous*, *Graminea*, *Pine*, *Artemisia*, *Palmae*, *Olea*, and *Typha* ^42^. No viruses were found in the dust particle samples ^42^. On the other hand, the studies showed that the concentration of metals in dust increases in dusty days ^35^. The main elements contained in the PM10 include (Na, Ca, Mg, Al, Fe) ^48, 57^. Among the carcinogenic metals in PM10 are Ni, Cr, As, and Cd ^57^. According to studies conducted in Jordan and Saudi Arabia, dust samples contained considerable amounts of radioactivity ^58, 59^. Among other compounds reported in the desert dust are cyanobacteria toxins ^51, 52^.

***Economic losses on health.*** The economic losses caused by the respiratory problems in Zabol, Iran, are estimated at about US$66 million ^32^. The total damage estimated from DSS to the health system in Iran and Iraq amounted to US$306 million ^33^.

### Planning and policymaking

***Education and training.*** The researchers emphasized that there should be health care recommendations for all affected individuals by DSS to reduce the vulnerability of population at risk, especially susceptible groups such as older persons, children, and cardiovascular and respiratory patients ^17, 36, 60^. Also, providing community-based training is an important role in the proper functioning of the people after receiving a warning message related to DSS ^49^.

***Research.*** Scientific findings focused on this issue that further epidemiological studies should be conducted to identify chemical compounds and microorganisms in PM10, the baseline incidence values for each country, and the potential effects of DSS on health, especially long-term effects ^18, 35, 37, 39, 43, 52– 55, 57, 61^. Moreover, there was no qualitative research among the studies reviewed.

***Compilation of new and local indexes of air quality.*** Based on the assessment of PM-related health risks, researchers found that it is necessary to design new standards for local ambient air quality in the EMR ^61, 62^. Only limited countries such as Saudi Arabia, Bahrain and Jordan have an air pollution index relevant to their country ^61, 63^.

***Comprehensive database development.*** To sum up the studies, provision of a comprehensive database of air pollutants and the effects of natural hazards, such as DSS, on health facilities is crucial for the benefit of policymakers and people ^57, 64^.

***Prediction and warning.*** Based on the findings of the studies conducted in EMR, the deployment of EWS such as the network-based integrated system of forecast and forewarning (DuSNIFF) can be a good base and framework for a timely warning to the population at risk ^49, 65^. Conversely, other researchers reported that there is no need for a warning to the emergency department of hospitals in the event of DSS occurrence ^53^.

### Risk assessment

***Use of hospital safety assessment tools.*** According to the assessment of Farsi Hospital Safety Index (FHSI), as a preparedness tool ^66, 67^, during different years in Iran, DSS was assessed as one of the highest probability of occurrence and health effects ^68^.

***Use models for health risk assessment of DSS.*** Based on the studies, health policymakers can find a better understanding of the health effects associated with PM10 peak times by using the findings of analytic models such as AirQ ^69^ and generalized additive model (GAM) ^18, 39^.

## Discussion

Preparedness for DSS in large-scale management and at the community level is one of the essential measures in the EMR. Provision of the necessary information such as the burden of diseases caused by DSS can be used in policy-making to focus on pre-disaster planning such as preparedness. The Hyogo Framework for Action (HFA) as an international strategy put emphasis on preparedness in order to produce effective response measures at all levels, to prioritize disaster risk reduction, and to reduce the background risk factors ^70^. Preparedness needs to be preserved and dynamic and ongoing efforts ^21^. Conducting research and producing scientific evidence relevant to disease burdens resulting from DSS can help to improve the health system preparedness for DSS ^71^. Given that health disorders caused by climate change affect the pattern and changing the burden of disease in the community, therefore, having basic guidelines on preparedness will make optimal use of resources in health service delivery ^72, 73^. The health managers in EMR must develop their readiness based on the recognition of the burden of acute and long-term diseases and different dimensions of phenomenon. DSS preparedness requires a clear understanding and assessment of the country's situation. Health centers also need to be prepared regarding the personnel, equipment, medicine, and infrastructure.

The trained people that have greater understanding are more aware of the risks of hazards and in the event of a disaster, they act more appropriately ^74, 75^. One of the top priorities of the Sendai Framework for disaster risk 2015–2030 is the understanding of disasters in all their dimensions to allow appropriate measures to be taken for disaster preparedness. This framework is the result of consultations and intergovernmental negotiations that were encouraged by the United Nations Office for Disaster Risk Reduction ^13^. Training, as one of the effective factors in the promotion of a disaster-preparedness culture, is essential in two levels: community-based education and training of health providers. Planning and doing regular drills in various scales is essential for the promotion of general and specialized education levels of the organizations and the people affected by this phenomenon. To enhance the understanding of disaster risk among managers and the community, awareness-raising programs should focus on capacity development through sharing previous experiences and lessons about preparedness for DSS. Also, the development of indigenous knowledge should be considered. For instance, in desert area, and not available to a mask, using of keffiyeh ^76, 77^ is recommended for protection from DSS.

With the purpose of planning for preparedness, regional policymakers must consider local considerations when using air quality indexes. Tsiouri *et al*. ^63^ and Murena ^78^ noted that different geographical regions have specific climatic conditions; therefore, this issue has an impact on atmospheric pollutants that affect human health as well as population responses to air pollution. As a result, the localization and adaptation of air pollution and its indicators can take place throughout the world. With the purpose of planning for preparedness, regional policymakers have to take into account that cannot be ignored local considerations when using air quality indexes ^63, 78^. To reduce the concerns, in light of valid regional evidence, the use of these standards should be reviewed.

Some studies in EMR ^18, 45, 50, 53, 54^ and other regions of the world have shown that there is no association between the occurrence of dust and increasing health problems ^79– 82^. However, it is important to conclude that with increasing frequency, intensity and geographic expansion of DSS, it is necessary to ensure a timely and valid warning to vulnerable populations and groups ^16, 83^. Provision of advanced and accurate warning systems requires continuous efforts to improve air quality modeling and prediction ^73^. Moreover, the results of various studies in the world show that early health warnings to vulnerable people about air pollution can reduce emergency visits to health centers through the reduction of outdoor activity during dusty days ^16, 83^.

The trans-boundary nature of DSS, unlike many natural hazards, is not limited to a specific geographic area; therefore, regional cooperation is needed to prepare for this phenomenon. In this regard, Kuwaiti scholars have suggested that it is essential to establish a regional committee ^54^. The health system of the countries involved in DSS give priority to the development of regional health memorandums of understanding (MoU). In the framework of these MoUs, countries can strengthen regional and global collaboration as with UN and WMO to transfer modern technologies for prediction of DSS occurrences, exchange of medical knowledge, allocation of financial and technical assistance, combat against desertification, share of information and successful practical experiences, to form a regional credit union, and to build training workshops.

### Strengths and limitations of the study

It can be noted, among the strengths of this study, the literature analysis performed with carefully assessing and have been done several times by the research team. Their research area was about Health in Disasters and Emergencies. The focus of this SR was on the EMR, as one of the most challenging areas, which has large DSS sources and creates regional health problems. A comprehensive study with this goal, so far, has not been conducted in this region. However, in this SR, only English articles related to EMR were included, the number of articles related to dust and health field was limited, and the full text of some studies was not accessible.

## Conclusion

The burden of diseases caused by dust in EMR shows the need to undertake measures for government preparedness to protect the health of affected. Given that DSS is a large-scale hazard, to gain preparedness, countries should move towards regional and international cooperation. The health system needs to develop a comprehensive plan of readiness to improve the effectiveness of the response measures. Also, regular exercises in all scales are a very important component. To increase public health recommended the development of dust-health EWS. Promotion of preparedness culture and the increase of public awareness about the effects of DSS through public media are suggested. In preparedness programs, the participation of the community is recommended. Health workers should receive regular training on cardiovascular and respiratory problems. Further quantitative and qualitative researches to identify the nature of DSS and adaptive factors can help bridge the gap between scientific findings and preparedness measures. Although we addressed preparedness in this study, there should be a comprehensive plan to manage the hazard and to consider all the loops of the disaster risk management cycle. In general, this study can help policy-makers of the health system in disaster risk management to identify factors that are effective in preparedness for DSS and to take the necessary preparedness measures.

## Data availability

All data underlying the results are available as part of the article and no additional source data are required.

## Reporting guidelines

A completed PRISMA checklist is available on figshare, DOI: https://doi.org/10.6084/m9.figshare.7581833 ^30^.

License: CC0 1.0 Universal.
