# Supplementary material for: Preparedness components of health systems in the Eastern Mediterranean Region for effective responses to dust and sand storms: a systematic review
Source: F1000Res. 2019 Feb 4;8:146. [Version 1] doi: 10.12688/f1000research.17543.1 (PMC6436192; doi:10.12688/f1000research.17543.1)
Supplement: Peer review history

# Peer review: Reviewer response for version 1

**DOI:** 10.5256/f1000research.19185.r46125

**Published:** 2019-03-25

**Authors:** Randah R. Hamadeh

**Affiliations:** 1 Department of Family and Community Medicine, College of Medicine and Medical Sciences, Arabian Gulf University, Manama, Bahrain

**General**

 The paper tackles an important topic on preparedness for dust and sand storm, a common phenomenon in the EMR that has not been much studied. The authors have done a systematic review on the topic and reviewed 30 articles in a comprehensive manner. I have some specific comments:****

****

**Abstract**

- Expand results section and reduce methods.
- Line 2: “Numerous health problems caused by DSS will be severely affected regions”: Something missing in the statement.
- Line 2, results: The preparedness components were divided into three and ten main categories and subcategories: Revise to “The preparedness components were divided into three main categories and ten subcategories”.

**Introduction**

- The introduction is concise and organized, however I feel that that the last lines “The findings of the study, with proposed recommendations, can directly help health policymakers in preparedness promotion for this phenomenon, pave the way for further studies, and add to the richness of the current knowledge” better be moved to discussion..

**Results**

- “Using of keffiyeh” in end of results, better to write what it means in brackets (Arab Headdress).

**Discussion**

- Move some parts of the discussion to the introduction where they are more relevant particularly from the first paragraph.
- The authors can also mention in the limitations that some articles might have been published in local scientific journals that are not indexed and thus were missed from the review.

I have read this submission. I believe that I have an appropriate level of expertise to confirm that it is of an acceptable scientific standard.

# Peer review: Reviewer response for version 1

**DOI:** 10.5256/f1000research.19185.r43965

**Published:** 2019-03-13

**Authors:** Mohamad Taghi Moghadamnia

**Affiliations:** 1 Department of Medical-Surgical Nursing, School of Nursing and Midwifery, Guilan University of Medical Sciences, Rasht, Iran

In this article, the authors survey preparedness components of health systems in the Eastern Mediterranean Region for effective responses to dust and sand storms using a systematic review approach. It addresses an important topic, especially the preparedness of health system in Eastern Mediterranean Region. Regarding climate change, increasing temperatures and dust storms, awareness of the readiness and response of health providers is important. In this systematic review, the authors attempted to study the studies carried out in this regard.

 Below are more specific comments:

- In the abstract, the authors have paid too much attention to the methods, and the results are very limited and incomplete.
- The keyword “reediness” is wrong, it would be better to write "readiness”.
- A large part of the material written at the beginning of the discussion section is related to the introduction, and it would be better to move that section. It is recommended that the authors, after a brief description of the subject framework, focused on the findings of their study and then compare them with the findings of others.
- In the conclusion, the authors must clearly and explicitly state the message of their study.

I have read this submission. I believe that I have an appropriate level of expertise to confirm that it is of an acceptable scientific standard.
